# Cyclic Esotropia Managed With Botulinum A Toxin Injections: A Report of Four Cases and Literature Review

**DOI:** 10.7759/cureus.46266

**Published:** 2023-09-30

**Authors:** Ahmed M Abdelaal, Amal Alhemidan, Reem A Alabdulqader, Laila H Jeddawi

**Affiliations:** 1 Pediatric Ophthalmology and Strabismus, Prince Sultan Military Medical City, Riyadh, SAU; 2 Pediatic Ophthalmology and Strabismus, King Fahad University Hospital, Imam Abdulrahman Bin Faisal University, Al Khobar, SAU; 3 Pediatric Ophthalmology and Adult Strabismus, Dhahran Eye Specialist Hospital, Dhahran, SAU

**Keywords:** strabismus, botulinum toxin a, extraocular muscle surgery, cyclic strabismus, cyclic esotropia

## Abstract

Cyclic strabismus is a rare entity and is unique in that patients follow variable but reliable time cycles where they alternate between orthotropia or “straight” eyes and strabismus, most commonly in the form of esotropia. Despite many theories on the underlying etiology and unique features of this diagnosis, none have been proven and its pathophysiology remains unknown. Four cases of cyclic esotropia diagnosed by pediatric ophthalmologists have been included in this report. The ages of the patients ranged from 10 months to eight years. The time duration from the onset of deviation to presentation to an ophthalmologist ranged from 1-52 weeks with three of the four patients presenting in the cyclic phase and the fourth presenting with a constant esotropia after a clear history and photographically documented cyclic esotropia for the preceding two months. All four patients were followed for periods ranging from one to four months to confirm their diagnosis or obtain multiple readings of the maximal deviation on the strabismic days before any intervention. The angle of esotropia when present ranged from 25 to 35 prism diopters and the cycle duration was 48 hours for all four cases (24 hours of esotropia followed by 24 hours of orthotropia). All patients were treated with botulinum toxin A injections to both medial recti, which resulted in an end to their cyclic deviation with excellent alignment obtained during follow-up periods ranging from 12-36 months for all cases. Cyclic esotropia is an elusive diagnosis that can be easily overlooked. When identified, classical treatment is usually extraocular muscle surgery targeting the largest angle of deviation. Many non-surgical treatments have been tried to no avail. However, in recent times, botulinum toxin A has been seen as a viable alternative.

## Introduction

Cyclic strabismus is a rare condition estimated to occur in one out of every 3,000-5,000 cases of strabismus and is characterized by periods of “straight” eyes or orthotropia alternating with large angle ocular deviations, most frequently esotropia, in cycles that classically occur every 48 to 120 hours [1]. Its rarity is such that it has been calculated that even ophthalmologists in the field of strabismus would need to practice for 14 years to encounter a single case of cyclic strabismus [2]. Despite its cyclic pattern of onset, cyclic strabismus will eventually convert to a permanent strabismus with time [3]. There have been various limited accounts describing this unusual form of strabismus, the vast majority being case reports or series due to the infrequency of the condition [2,4-14].

Theories and hypotheses of the etiology underlying cyclic strabismus have been proposed with gradual loss of control of latent strabismus, incomplete cerebral dominance with interhemispheric rivalry, neuromyotonia and dysfunction of the biologic circadian clock mechanism all postulated but not proven [2,3,15-18]. Even an investigator who dedicated a tremendous amount of his time and efforts to studying the biological clock mechanism and proved their relationship and roles in many aspects of biological human functions including behavior and metabolism, could not find an explanation for the circadian pattern of cyclic strabismus [16]. Even in the presence of cases being reported following visual compromise, trauma, strabismus surgery, and central nervous system pathology, the underlying etiology of cyclic strabismus remains unknown and its etiology is still considered to be idiopathic [4,6,8,12,19-21].

Despite its bizarre presentation and idiopathic nature, surgery in the form of bilateral medial rectus recession (BMR) based on the deviation measured on the strabismic days is the established and classical standard treatment, with satisfactory postoperative ocular alignment and most importantly, no reversal of the original deviation due to an overcorrection on the “straight” or orthotropic days of the cyclic strabismus [2,3,5,16,17,22].

Due to the rarity of cyclic strabismus and the paucity of literature containing two or more cases, we present this report of four patients with cyclic esotropia managed with botulinum toxin A injections to contribute to the scarce evidence regarding treatment approaches for this poorly understood condition. To our knowledge, this represents the first report of cyclic strabismus cases from the Gulf and Middle East region.

## Case presentation

We present four cases of cyclic esotropia along with their follow-up results after treatment using botulinum toxin A, along with a comprehensive review of relevant literature including theories and innovative treatments. Our cases were obtained by asking three senior pediatric ophthalmology and strabismus consultants to review their strabismus cases for the diagnosis of cyclic esotropia and cyclic strabismus. Informed written consent was obtained for all four participants and all patients were treated in accordance with the Declaration of Helsinki. All procedures were performed under general anesthesia in the operating rooms after sterilization and draping by grasping the medial rectus muscle using forceps and passing the needle intramuscularly through the conjunctiva and tenon capsule and injecting 5 IU of botulinum toxin A.

Case 1

**Figure 1:**
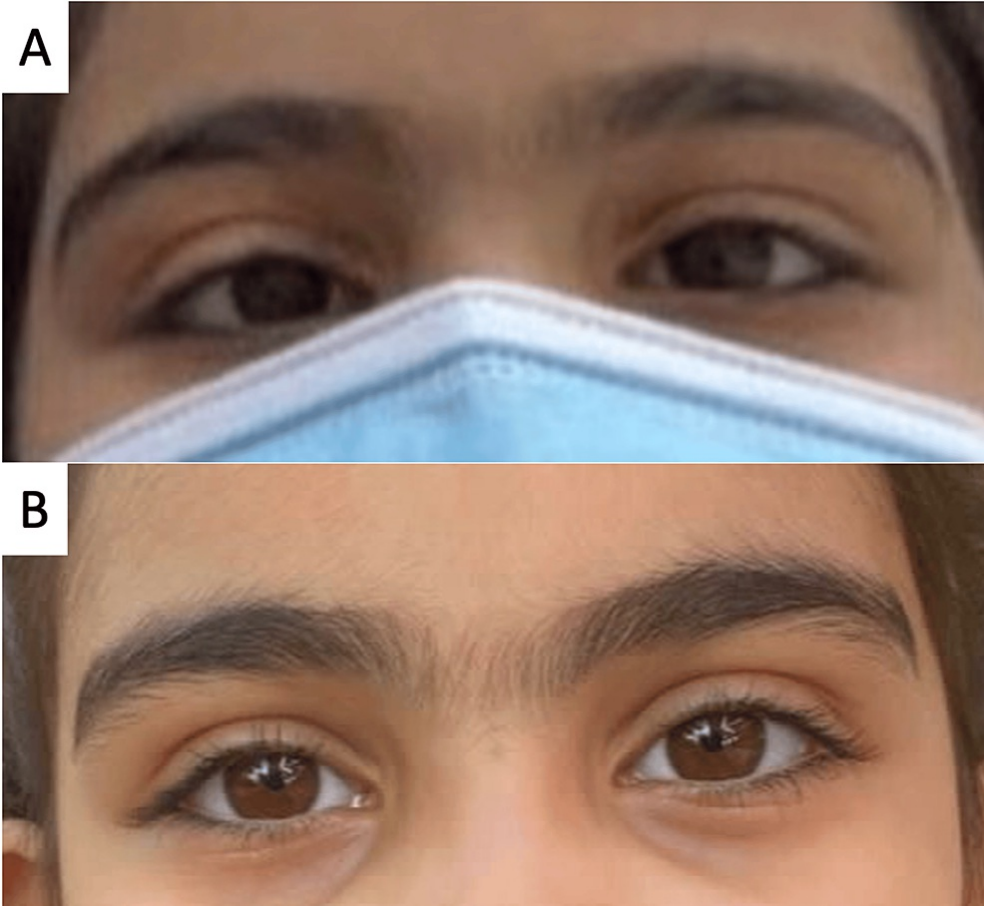
Case 1 (A) Pre-operative left esotropia; (B) Six months after botulinum toxin A injection showing the absence of any ocular deviation

A medically free eight-year-old girl presented to her ophthalmologist two months after starting to experience inward eye deviations of the left eye. Her deviation had started in a cyclic manner with 24 hours of esotropia followed by 24 hours without any deviation resulting in 48-hour cycles. During her episodes of esotropia, she developed symptomatic diplopia and would patch her left strabismic eye to avoid double vision. During this period, her conscious and anxious parents documented the deviation with multiple photographs on different days and shared it with the ophthalmologist. They denied any fever, headache, or other neurological symptoms or antecedent trauma. After two months of cyclic esotropia and patching the left eye on strabismic days, her deviation became constant. At the time of presentation, she had 20/20 unaided visual acuity (VA) in both eyes and an esotropia measuring 35 prism diopters (PD) at near and distance without correction (Figure 1A). Dilated funds examination was normal and cycloplegic refraction was unremarkable. A review of photographs provided by the parents revealed a cyclic onset with cycles occurring every 48 hours over the preceding two months. Consequently, the clinical diagnosis of cyclic esotropia was made and three months following the onset of her complaint, the patient was given injections of 5 IU of botulinum toxin A into both medial recti muscles. On follow-up at two months and 6 months, she was orthotropic by both Hirschberg and cover testing and remained so till the writing of this report two years later (Figure 1B). No changes in VA or refraction occurred from her pre-operative examination throughout her follow-up.

Case 2 

**Figure 2:**
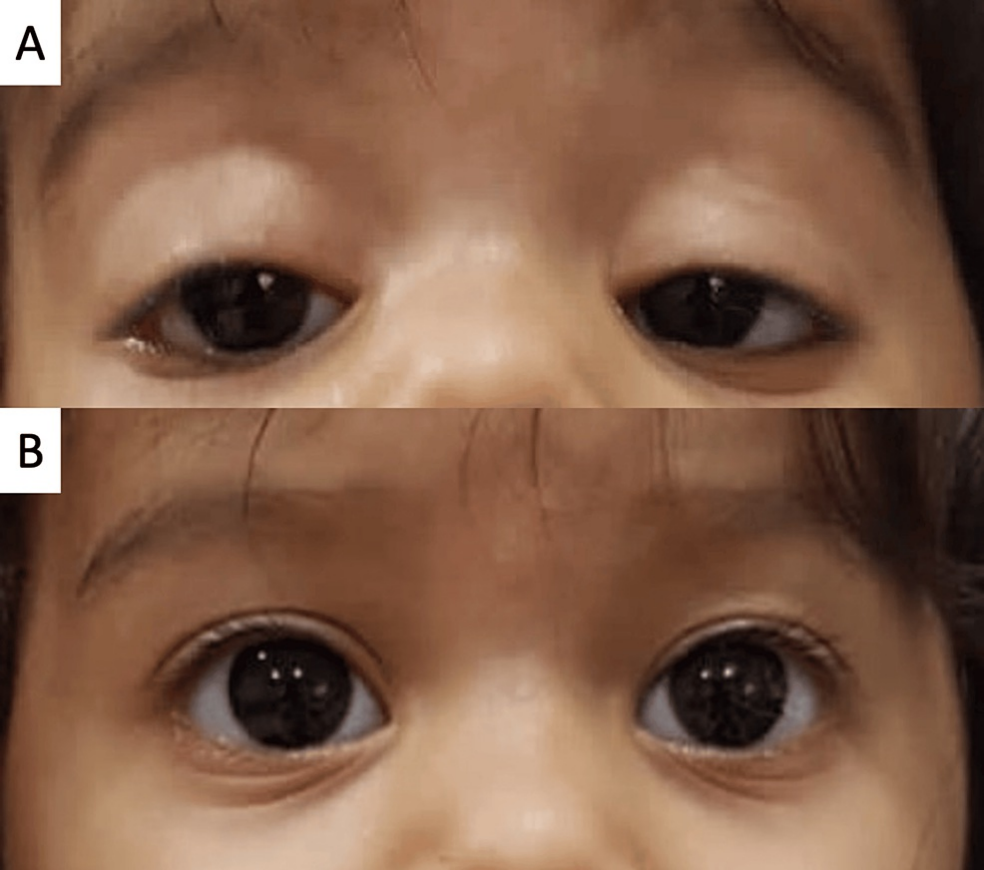
Case 2 (A) Left esotropia and bilateral ptosis five days after botulinum toxin A injection; (B) Six months after botulinum toxin A injection showing the absence of any ocular deviation

A 10-month-old girl, not known to have any medical complaints was referred to with a one-week history of isolated inward eye deviation. She had originally presented to a pediatric neurologist who found no neurological abnormalities on history or examination except for her eye deviation. A neurological-ophthalmic evaluation and an MRI of the brain and orbits were also performed and both were unremarkable. When seen by her pediatric ophthalmologist, the examination showed equal vision of fixation and following in both eyes with the absence of any deviation on orthoptic testing, and an otherwise unremarkable exam including a cycloplegic refraction and dilated fundus evaluation. The family was adamant about the occurrence of a manifest deviation over the past week and was advised to bring the child back to the clinic once they noted any deviation. She was seen on the next day and her orthoptic testing revealed freely alternating esotropia between both eyes of 25 PD at near and distance without correction. The repeated ophthalmic evaluation showed central, steady, and maintained vision in both eyes, and repeat dilated fundoscopy and cycloplegic refraction remained unchanged. As a result, the clinical diagnosis of cyclic esotropia was made. Two months after her initial presentation with multiple evaluations to document the cyclic pattern of her esotropia and obtain multiple measurements of her angle to assess the largest angle of esotropia that revealed no significant changes, she underwent bilateral medial rectus injections of 5 IU of botulinum toxin A. At her two-week follow-up, she had moderate ptosis in both eyes and a small angle alternating exotropia. At six weeks following botulinum toxin A injection, there was no more ptosis and Hirschberg testing and cover testing revealed no deviation, she maintained these findings to her last follow-up visit 12 months later (Figure 2).

Case 3

**Figure 3:**
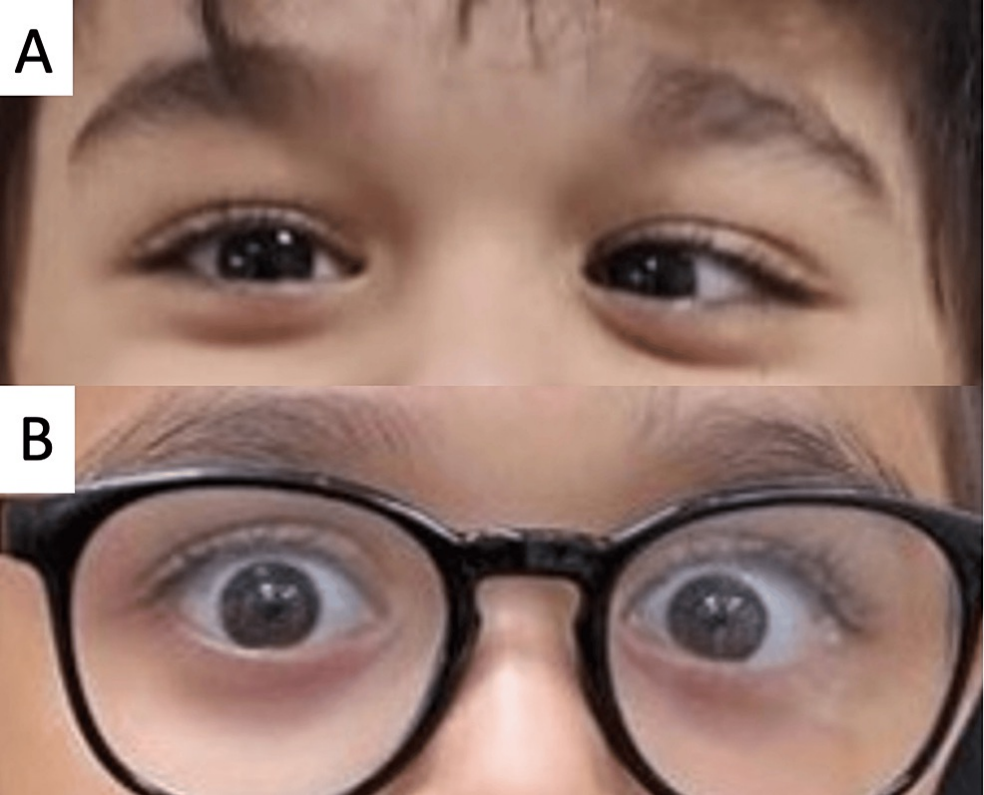
Case 3 (A) Pre-operative left esotropia; (B) absence of any ocular deviation 24 months after botulinum toxin A injection

A healthy three-year-old boy was brought by his family due to an on-and-off eye deviation for the past 12 months. There was no history of fever, headaches, neurological or other systemic symptoms preceding or accompanying the deviation. Examination revealed unaided visual acuity of 20/30 in each eye and an orthoptic evaluation showed 35 PD of left esotropia with preference for the right eye. The remainder of his examination including a retinal evaluation were unremarkable except for a cycloplegic refraction that showed astigmatism of -2.00 in both eyes. As such the child was given spectacles for the cylindrical refractive error and was followed for the ensuing two months to assess visual acuity with spectacle correction and response to part-time occlusion of the favored right eye. During the four consequent visits, his corrected visual acuity reached 20/20 in both eyes and the same 35 PD of left esotropia was present at near and distance with and without spectacle correction. However, one day the family presented to the clinic without an appointment claiming his eyes were now straight. The child was re-assessed completely on that day and his orthoptic exam now showed orthotropia with no ocular deviation at near or distance with or without his correction, all other findings were the same including aided visual acuity, fundoscopy, and cycloplegic refraction. For the next month, the child had daily photographic documentation of his ocular alignment and was re-examined on both strabismic and straight days before a definitive diagnosis of cyclic esotropia was made. After a thorough discussion of the risks and benefits of available treatment options, the child had an injection of 5 IU of botulinum toxin A into both medial recti with excellent results. Orthotropia was achieved at one, six, and 12-month follow-up visits using Hirschberg and alternate cover testing, and excellent alignment was maintained up to the last follow-up 24 months after the procedure (Figure 3).

Case 4

**Figure 4:**
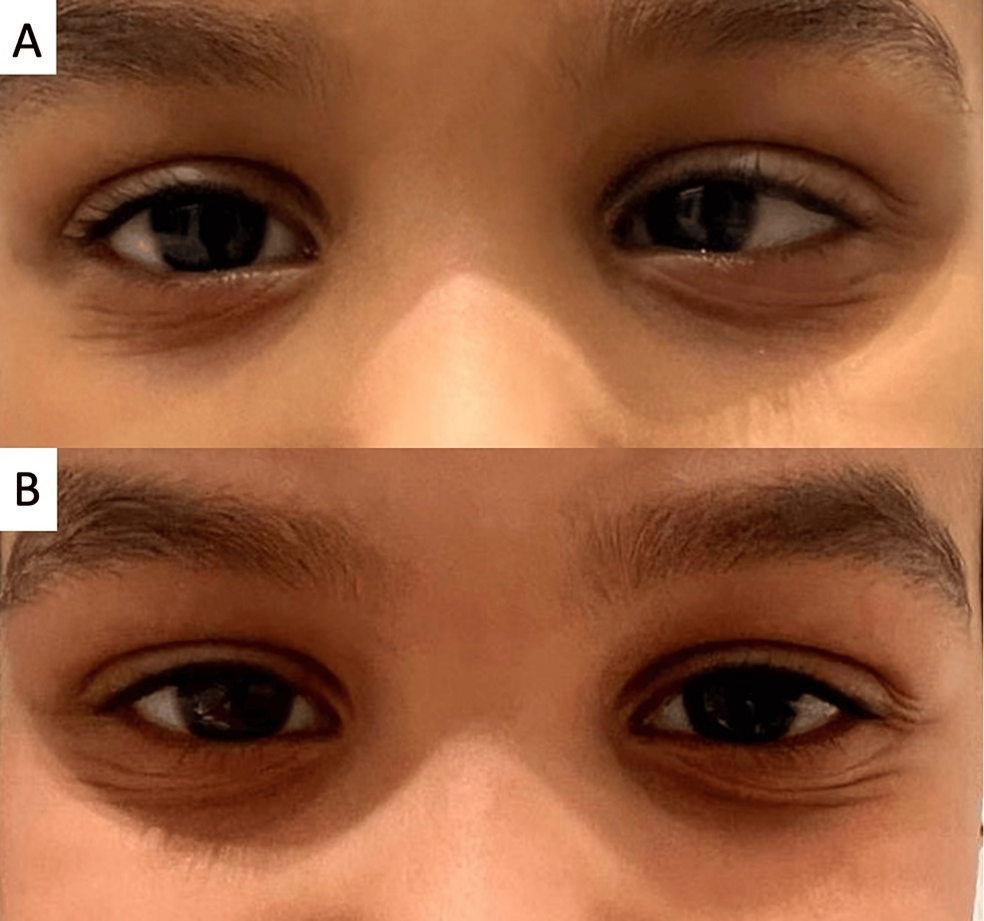
Case 4 (A) Pre-operative left esotropia; (B) Six months after botulinum toxin A injection, showing the absence of any ocular deviation

A five-year-old, medically free girl was brought by her parents after they had noticed transient inward deviation of her left eye for the past two months that had been occurring every other day and lasting 24 hours, followed by 24 hours without any ocular deviation. There were no occurrences of trauma, fever, or neurological symptoms preceding or at the time of presentation. Examination revealed 20/20 unaided visual acuity and 30 PD of alternating esotropia that was more commonly occurring in the left eye at both near and distance without refractive correction. Other examinations including dilated funduscopy and cycloplegic refraction were unremarkable. She was followed and observed for four months after presentation on both strabismic and “straight” days to confirm her diagnosis of cyclic esotropia and obtain multiple accurate measurements of her largest angle of deviation on strabismic days. No change in refraction or the angle of deviation when present was noted during follow-up until the child's reluctant parents agreed to have an intervention done for her cyclic strabismus. Due to the reluctance of the family to undergo a surgical procedure, treatment was offered in the form of botulinum toxin A injection to both medial recti, and excellent alignment was obtained. After one, six, and 12 months of follow-up, orthotropia was maintained as tested by Hirschberg and cover testing and no complications were encountered in the postoperative period (Figure 4).

Table 1 summarizes patients' characteristics, presentation, examination findings, and management as well as the follow-up results for all cases.

**Table 1:** Summary of cases showing patient characteristics, exam findings, management, and follow-up period and results BCVA =Best corrected visual acuity, VAsc = Uncorrected visual acuity, OD = Oculus dexter (right eye), OS = Oculus sinister (left eye), PD = Prism diopters, ET = Esotropia, CSM = Central, Steady, Maintained

	Age	Gender	BCVA on Deviation Days	Deviation Angle	Cycle duration	Cycloplegic Refraction	Treatment	Follow-up Period	Follow-up Result
Case 1	8 years	Female	VAsc OD 20/20 - OS 20/20	35 PD ET	48 hours	OD Plano - OS Plano	Bi-Medial Rectus Botulinum Toxin A Injection	24 months	Orthotropia
Case 2	10 months	Female	OD CSM - OS CSM	25 PD ET	48 hours	OD +1.50 - OS +1.50	Bi-Medial Rectus Botulinum Toxin A Injection	12 months	Orthotropia
Case 3	3 years	Male	VAsc OD 20/30 - OS 20/30	35 PD ET	48 hours	OD +0.25 -2.00 x015 - OS +0.25 -2.00 x155	Bi-Medial Rectus Botulinum Toxin A Injection	24 months	Orthotropia
Case 4	5 years	Female	VAsc OD 20/20 - OS 20/20	30 OD ET	48 hours	OD +1.50 -0.50 x020 - OS +1.50 -0.50 x020	Bi-Medial Rectus Botulinum Toxin A Injection	12 months	Orthotropia

## Discussion

The most common type of cyclic strabismus is nonaccommodative cyclic esotropia; however, congenital, accommodative, and consecutive forms of cyclic esotropia have also been reported [2,10]. Despite its cyclic nature at onset, when left untreated, cyclic strabismus is believed to eventually convert to permanent strabismus with time [2,3]. However, a case of cycling every 48 hours for 15 years before abating following surgical intervention has been described [23].

All cases included in our study were cyclic esotropia, which is in keeping with reports in the literature that it is the most frequently encountered form [2,10]. None of our patients had a high AC/A ratio associated with their esotropia which had been reported in a few cases in the past. Due to this finding and the observation that no patients had accompanying cyclical changes in accommodation or pupil size, we believe the most likely explanation for cyclic strabismus to be related to neurological disturbances originating from the brainstem. No laterality preference has been reported before for cyclic esotropia. Three of the four patients in the current report manifested their deviations in the left eye and one had freely alternating esotropia between both eyes.

Our patients included one male and three females ranging in age from 10 months to eight years of age. Of the four cases, cyclic strabismus had been present for a time that ranged between one and 52 weeks. The usual behavior of the cycles observed in cyclic esotropia is for esotropia to manifest on alternate days (every 24 hours) in the setting of “straight” orthotropic eyes during the other day of the cycle (24 hours), giving the classical 48-hour cycle that is most often described. However, variable cycle durations of three to five days have been reported [1,9]. A recent case report described the shortest cycle to date consisting of 12 hours of orthotropia alternating with 12 hours of esotropia for a complete cycle of 24 hours [24]. In the present report, the cycle duration was 48 hours for all cases, supporting the most commonly encountered cycle duration reported in the literature.

Attempted non-surgical treatments for cyclic strabismus had previously included baclofen, di-isopropyl fluorophosphate, ecothiopate iodide, and meiotic agents without success [2,5,25]. However, a more recent report by Voide et al. has advocated non-surgical treatment using prisms for a latent deviation found on "straight days" [26]. In that case, an esophoria was treated with a 6 base-out press on Fresnel prism for one month resulting in a break of the patient’s cyclic strabismus with no recurrence at 24 months of follow-up. The authors believed their success was due to restoration of compensation and normal binocular function and recommended non-invasive therapy using prisms to be tried as a first-line therapy, with surgical options used for non-responsive cases [26].

The classical standard treatment for cyclic esotropia is BMR aiming to correct the largest measurable deviation present on strabismic days, which yields excellent postoperative alignment without overcorrection on the previously “straight” days [2,3,5,16,17,22]. However, there have been relatively modern reports of successfully managing cyclic esotropia using botulinum toxin A [27-29]. The mechanism by which botulinum toxin A treats cyclic esotropia is not known, but the theory is that it acts by strengthening the antagonist muscle, with this strengthening lasting even after its duration of action is completed, resulting in a new equilibrium [28]. The advantages put forth for this less invasive approach of treatment include minimal scarring of ocular tissues and conservation of extraocular muscles for future surgeries if required [30]. The disadvantages include unpredictable responses that may lead to transient exotropia and ptosis [30]. In our report, all four patients were treated with botulinum toxin A with excellent results. All had angles ranging between 25 and 35 PD and received 5 IU into both medial recti. With follow-up periods ranging from one to two years, all have restored alignment. From our experience and excellent results, we advocate the use of botulinum toxin A without hesitation as an alternate treatment to surgery, especially in the setting of reluctant parents, after a thorough discussion on the expected results and possible complications. We found that botulinum toxin A can provide predictable and long-term alignment in patients with cyclic esotropia and shows no clinical inferiority to extraocular muscle surgery.

With some having reservations over the true and long-term effectiveness of nonsurgical treatment with prisms and less invasive surgical options including botulinum toxin A that may exist due to limited reports, many experts still elect to perform extraocular muscle surgery as it has proven to practically always treat cyclic esotropia [5,19,20,25,31]. From our experience managing four cases with adequate durations of follow-up, a thorough discussion of all treatment options should be conducted with patients and their families before more invasive procedures are performed. It is well established that surgery in the form of BMR provides excellent success rates and very predictable results [26-28,32,33]. However, from our experience and the increasing body of evidence on botulinum toxin A, we believe it may be offered as a first-line of invasive intervention before surgery, and the patient and family allowed to consider the risks and benefits of both options equally.

## Conclusions

As cyclic esotropia remains an uncommon form of strabismus rarely encountered by most ophthalmologists, increased awareness of the condition is warranted to avoid misdiagnosis and unnecessary investigations as well as familiarizing ophthalmologists of possible alternatives to extra-ocular muscle surgery. It is important to analyze existing results where less invasive alternative treatments have been done and compared to standard extraocular muscle surgery before concluding a similar efficacy. Larger, controlled, and comparative studies would also give much more solid conclusions. However, from our limited experience, we found treatment with botulinum toxin A for the largest angle of esotropia on strabismic days for patients who warrant an invasive procedure to be safe and successful at treating cyclic esotropia.

## References

[1] Costenbader FD, Mousel DK (1964). Cyclic esotropia. Arch Ophthalmol.

[2] Helveston EM (1973). Cyclic strabismus. Am Orthopt J.

[3] Caputo AR, Greenfield PS (1978). Cyclic esotropia. Ann Ophthalmol.

[4] Cole MD, Hay A, Eagling EM (1988). Cyclic esotropia in a patient with unilateral traumatic aphakia: case report. Br J Ophthalmol.

[5] Metz HS, Jampolsky A (1979). Alternate day esotropia. J Pediatr Ophthalmol Strabismus.

[6] Stager D Jr, Thyparampil PJ, Stager DR Sr (2010). Cyclic exotropia in a child. J AAPOS.

[7] Hutcheson KA, Lambert SR (1998). Cyclic esotropia after a traumatic sixth nerve palsy in a child. J AAPOS.

[8] Gadoth N, Dickerman Z, Lerman M, Lavie P (1981). Cyclic esotropia with minimal brain dysfunction. J Pediatr Ophthalmol Strabismus.

[9] Lee JY, Seok S, Oh SY (2014). A case of cyclic esotropia with menstrual cycle. Acta Ophthalmol.

[10] Kee C, Hwang JM (2014). Accommodative esotropia decompensated to cyclic esotropia in a 6-year-old boy. J AAPOS.

[11] Ngo CS, Araya MP, Kraft SP (2015). Cyclic strabismus in adults. J AAPOS.

[12] Troost BT, Abel L, Noreika J, Genovese FM (1981). Acquired cyclic esotropia in an adult. Am J Ophthalmol.

[13] Garg SJ, Archer SM (2007). Consecutive cyclic exotropia after surgery for adult-onset cyclic esotropia. J AAPOS.

[14] Murthy R, Hegde S (2009). Acquired cyclic exotropia and hypotropia. J AAPOS.

[15] Metz HS (2003). Light and the circadian clock. J AAPOS.

[16] Richter CP (1968). Clock-mechanism esotropia in children. Alternate-day squint. Johns Hopkins Med J.

[17] Parlato CJ, Nelson LB, Harley RD (1983). Cyclic strabismus. Ann Ophthalmol.

[18] Roper-Hall G, Cruz OA, Espinoza GM, Chung SM (2013). Cyclic (alternate day) vertical deviation--possible forme fruste of ocular neuromyotonia. J AAPOS.

[19] Muchnick RS, Sanfilippo S, Dunlap EA (1976). Cyclic esotropia developing after sttstrabismus surgery. Arch Ophthalmol.

[20] Uemura Y, Tomita M, Tanaka Y (1977). Consecutive cyclic esotropia. J Pediatr Ophthalmol.

[21] Pillai P, Dhand UK (1987). Cyclic esotropia with central nervous system disease: report of two cases. J Pediatr Ophthalmol Strabismus.

[22] Lai YH, Fredrick DR (2005). Alteration of cyclic frequency by botulinum toxin injection in adult onset cyclic esotropia. Br J Ophthalmol.

[23] Souza-Dias C, Kushner BJ, Rebouças de Carvalho LE (2018). Long-term follow-up of cyclic esotropia. J Binocul Vis Ocul Motil.

[24] Paciuc-Beja M, Galicia-Alfaro VH, Retchkiman-Bret M (2019). Unusual presentation of an uncommon disease: 24-hour cyclic esotropia. Case Rep Ophthalmol Med.

[25] Helveston EM (1976). Surgical treatment of cyclic esotropia. Am Orthopt J.

[26] Voide N, Presset C, Klainguti G, Kaeser PF (2015). Nonsurgical treatment of cyclic esotropia. J AAPOS.

[27] Jones A, Jain S (2014). Botulinum toxin: a novel treatment for pediatric cyclic esotropia. J AAPOS.

[28] Wipf M, Bok-Beaube C, Palmowski-Wolfe A (2018). Botulinum toxin for the treatment of cyclic strabismus in children: three case reports. Klin Monbl Augenheilkd.

[29] Jang IE, Davis JA, Epley KD, Cabrera MT (2020). Botulinum toxin for the treatment of cyclic esotropia in a child with Chiari type I malformation. J AAPOS.

[30] Mahan M, Engel JM (2017). The resurgence of botulinum toxin injection for strabismus in children. Curr Opin Ophthalmol.

[31] Merrill K, Anderson J, Watson D, Areaux RG Jr (2019). A cluster of cyclic esotropia: white matter changes on MRI and surgical outcomes. J Pediatr Ophthalmol Strabismus.

[32] Firth AY, Burke JP (2007). Botulinum toxin for the treatment of acute-onset concomitant esotropia in Chiari I malformation. Br J Ophthalmol.

[33] Imes RK, Quinn TA (2001). Acute comitant esotropia in Chiari 1 malformation. Ophthalmology.

